# Beyond PD-1: The Next Frontier for Immunotherapy in Melanoma

**DOI:** 10.3389/fonc.2021.640314

**Published:** 2021-03-01

**Authors:** Anjali Rohatgi, John M. Kirkwood

**Affiliations:** Hillman Cancer Center, University of Pittsburgh Medical Center, Pittsburgh, PA, United States

**Keywords:** melanoma, checkpoint inhibition/blockade, pathogen recognition receptor (PRR), cytokines, TLR (Toll-like receptors)

## Abstract

The advent of first and second-generation immune checkpoint blockade (ICI) has resulted in improved survival of patients with metastatic melanoma over the past decade. However, the majority of patients ultimately progress despite these treatments, which has served as an impetus to consider a range of subsequent therapies. Many of the next generation of immunotherapeutic agents focus on modifying the immune system to overcome resistance to checkpoint blockade. ICI resistance can be understood as primary, or acquired—where the latter is the most common scenario. While there are several postulated mechanisms by which resistance, particularly acquired resistance, occurs, the predominant escape mechanisms include T cell exhaustion, upregulation of alternative inhibitory checkpoint receptors, and alteration of the tumor microenvironment (TME) into a more suppressive, anti-inflammatory state. Therapeutic agents in development are designed to work by combating one or more of these resistance mechanisms. These strategies face the added challenge of minimizing immune-related toxicities, while improving antitumor efficacy. This review focuses upon the following categories of novel therapeutics: 1) alternative inhibitory receptor pathways; 2) damage- or pathogen-associated molecular patterns (DAMPs/PAMPs); and 3) immune cell signaling mediators. We present the current state of these therapies, including preclinical and clinical data available for these targets under development.

## Introduction

The use of checkpoint inhibitors in melanoma has dramatically changed treatment options for patients with melanoma. Prior to 2011 and the FDA approval of ipilimumab, standard of care options included chemotherapy and high dose IL-2, and in the adjuvant setting, high-dose interferon alpha-2, all of which were associated with limited efficacy and significant toxicity. Targeted therapy with BRAF and MEK inhibition has also been approved for the 50% of melanoma patients with activating BRAF mutations. Despite a promising overall response rate and evidence of durable responses for some, the majority of patients with advanced melanoma have ultimately exhibited progression of disease on or after checkpoint blockade (1). Further, while the majority of patients tolerate therapy, there is risk of significant and sometimes fatal toxicity.

Significant effort has been put into finding ways to re-sensitize tumors after immunotherapy resistance has developed as well as alternative strategies for checkpoint blockade. In fact, the number of trials of combination inhibitors has increased significantly each year, though success of this approach remains to be seen (2). In this review, we will profile some of the most promising strategies in broad categories, including 1) alternative checkpoint receptors 2) DAMPs/PAMPs and 3) immune cell signaling modulators of the TME.

In order to understand the rationale for many of these novel therapies, mechanisms of anti-PD-1/PDL-1 resistance need to be discussed. Resistance is characterized as primary or secondary. The Society for Immunotherapy of Cancer taskforce recently published consensus guidelines to define these terms (3). Primary resistance is defined as progression of disease or at best stable disease for less than 6 months for patients who received a minimum of 6 weeks of therapy. Secondary resistance is defined as nonresponse with progression of disease after initial response to therapy with at least complete response (CR), partial response (PR) or stable disease (SD) of greater than 6 months duration. The mechanisms of resistance to anti-PD-1 therapy are postulated to be diverse, but this remains an area of exploration. In particular, identifying which mechanism or mechanisms are responsible for disease progression in individual patients is an area of ongoing interest.

A full review of immunotherapy escape mechanisms is outside the scope of this review; this topic has been reviewed extensively elsewhere and is summarized briefly here (4–7). Mechanisms attributed to resistance include a lack of target tumor neoantigens, or impaired antigen presentation in the tumor. Further, a lack of tumor immune cell infiltration described as a “cold” tumor, within which the non-inflamed tumor lacks the effector T cell populations that are the basis of benefit from ICI has been reported. Further, impairment of IFNγ secretion or signaling or other inflammatory cytokine responses can lead to resistance (8). This is often accompanied by presence of other types of suppressive immune cells, including M2 macrophages, T regulatory cells (Tregs), and myeloid derived suppressor cells (MDSCs). Alternative checkpoints that may govern the antitumor function of T cells, such as LAG3, TIM3 and other inhibitory receptors are also discussed here, as they can lead to reduction of antitumor cytotoxicity of T cells and are observed in exhausted T cells after chronic antigen stimulation. We will focus on strategies for which clinical data from ongoing trials are anticipated.

A combinatorial approach with ICI has been favored for many of these therapies, both because of limited efficacy seen thus far with many of the single agents being explored, and also because of postulated mechanisms of resistance to immunotherapy. For example, an alternative inhibitory receptor may reduce T-cell exhaustion, but may not be sufficient to promote T effector cytotoxicity without the concurrent administration of anti-PD-1. An example of successful combinatorial therapy is seen with dual-checkpoint inhibition using nivolumab and ipilimumab, as seen in several disease types including melanoma, lung cancer, and renal cell carcinoma. However, increases in response rates compared to ipilimumab monotherapy have come at the expense of increased toxicity (9). Where available, efficacy and toxicity with combination therapy are reported in this review.

## Alternative Checkpoint Receptor Pathways

Additional inhibitory checkpoint receptors are being explored as a potential avenue for single agent or combined therapy with anti-PD-1. Many of these receptors were identified in the setting of chronic viral infection, which leads to T cell exhaustion and unresponsiveness to stimuli. Therapies targeting inhibitory receptors are postulated to augment anti-tumor response, perhaps by reversing T cell exhaustion. Most of these are monoclonal antibodies that act *via* inhibitory receptors to relieve inhibition of T cell activity. This review will focus on effector T cell receptors, however it is worth noting that additional strategies for targeting other suppressive immune cell actors including MDSCs and Tregs are under development.

### Lymphocyte Activation Gene-3

Lymphocyte activation gene-3 (LAG3) (CD223) is a type I membrane protein found on the surface of activated T cells, T regulatory cells, NK cells, and plasmacytoid dendritic cells (10). LAG3 demonstrates homology to the costimulatory membrane protein CD4 and binds major histocompatibility complex II (MHCII) (11). LAG3 expression on T cells is upregulated after continued antigenic stimulation, and often co-expressed with additional inhibitory receptors such as PD-1 and TIGIT (12). LAG3 can also be proteolytically cleaved, releasing the external portion to become soluble LAG3, the role of which remains unclear (13). LAG3 has multiple functions in suppressing the immune response, as elucidated by murine knockout models. First, LAG3 decreases CD4+ T cell proliferation and secretion of inflammatory cytokines such as IL-2, IFNγ, and TNFα (14). LAG3 also affects the development of memory CD4+ T cells (15). LAG3 promotes the suppressive activity of Tregs, and inhibition of LAG3 can reduce Treg formation (16, 17). Similar immunosuppressive properties of LAG3 have been observed in human tumor samples (18). These data support LAG3 as an additional promising clinical immunotherapy target.

LAG3-targetting agents are currently under clinical investigation from several companies. Soluble LAG3 peptide (IMP321; eftilagimod alpha) has been tested in several early phase clinical trials for patients with solid tumors, both as monotherapy and as an adjuvant to vaccine development. In a phase I/IIa trial in resected melanoma, IMP321 was used as an adjuvant with a peptide vaccine in which the primary objective was to evaluate the T cell response and toxicity of the therapy (19). Indeed, CD8+ and CD4+ T cell responses were induced in the majority of patients. Additional studies have been conducted in combination with gemcitabine in patients with pancreatic cancer with a best response of stable disease (20), and in combination with paclitaxel for breast cancer with an objective tumor response rate of 50% (partial responses in 15/30 patients) (21). Partial responses have also been seen in combination with pembrolizumab in head and neck carcinoma in a phase II trial (22). Ongoing combinatorial strategies for IMP321 are under investigation.

Further, a number of anti-LAG3 monoclonal antibodies is under development, including BMS-986016 (relatlimab), LAG525, TSR-033, REGN3767, and MK-4280. These antibodies are under therapeutic evaluation in patients with melanoma, both as monotherapy and in combination with anti-PD-1. Further, they are being explored in the neoadjuvant and metastatic settings. Recruitment is ongoing for the majority of these studies. Preliminary data from NCT01968109, reporting the results of relatlimab plus nivolumab in melanoma patients who have received prior immunotherapy was reported at ESMO 2019 with an ORR of 11.5% (23). In a Phase I study of advanced solid tumors, LAG525 was given with or without spartalizumab resulting in the majority of patients discontinuing treatment for progressive disease (79% and 67% respectively) (24). Efficacy was reported as 11 PRs and 1 CR in the combination arm. Together, these data demonstrate less success with second line LAG3 inhibition than had been anticipated, but larger phase II studies in the treatment refractory and treatment naïve setting are needed to assess the potential role of this agent and are forthcoming.

### T Cell Immunoglobulin and Mucin-Domain Containing-3

T cell immunoglobulin and mucin-domain containing-3 (Tim-3) is a type I transmembrane protein found on the surface of T cells, NK cells, dendritic cells and macrophages (25). Tim-3 has several ligands including galactin-9, engagement of which results in cell death of Th1 cells (26). Other ligands include ceacam1, which may stabilize Tim-3 on the cell surface, and HMGB1 and phosphatidylserine (27). Tim-3 is also a marker of exhausted T cells, and is often co-expressed on CD8+ T cells with PD-1 (28). Tim-3 is associated with decreased inflammatory cytokine production of IFNγ, and can also enhance the immunosuppressive activity of Foxp3 Tregs, (29) (30). Tim-3 can also contribute to the suppressive TME by promoting the generation of MDSCs (31). In humans, Tim-3 is implicated in autoimmunity as well as chronic viral infections (29, 32). Patients whose tumors exhibit high levels of Tim-3 expression are more likely to have worse prognosis in several tumor types (27).

Similar to LAG3, Tim-3 is an attractive clinical target and several monoclonal antibodies targeting Tim-3 are under investigation including MGB453, TSR-022, Sym023, BGBA425, RO7121661, ICAGN02390, LY3321367, and BMS-986258. Ongoing trials with these antibodies were recently summarized in the review by Acharya, et al (33). Clinical data is forthcoming. LY3321367 alone, or with an anti-PD-L1 therapy, did not produce any dose-limiting toxicities, and was associated with >20% tumor regression (1 PR) in the monotherapy arm (NCT03099109) (34). For patients with NSCLC and melanoma treated with prior anti-PD-1/PD-L1, MBG453 was given with spartalizumab in a phase II study. Of the 33 patients in that study, 15.2% were being treated at the time of abstract presentation, with the remainder discontinuing study due to progressive disease. Grade 3/4 adverse effects including pruritis, amylase and lipase elevation, increased ALT were noted (35). As with the studies of LAG3, mature data and larger studies are in development.

### T Cell Immunoreceptor With Immunoglobulin and ITIM Domain

T cell immunoreceptor with immunoglobulin and ITIM domain (TIGIT) is another inhibitory receptor on T cells, as its name implies. TIGIT is a found on activated CD8+ and CD4+ T cells, NK cells, Tregs and T follicular cells (36). TIGIT binds to multiple ligands including CD155 and CD112, for which binding competes with the co-stimulatory receptor CD226/DNAM-1 (37). TIGIT is highly expressed in tumor samples and T cells that also express PD-1, suggesting a role in T cell exhaustion (38). In murine models of CT26 colorectal carcinoma, monotherapy with anti-TIGIT therapy did not effect tumor growth; however, when introduced with anti-PD-1 it resulted in a reduction of tumor growth (38). Further, this combination increased percent of tumor-infiltrating IFNγ+ CD8+ T cells (38). The anti-tumor effect of TIGIT may also be mediated by NK cells and enhanced by IL-15 (39).

A number of anti-TIGIT monoclonal antibodies are under development as listed in **Table 1**. A recent review by Chauvin, et al. lists ongoing Phase I/II clinical trials involving TIGIT, which are primarily being conducted with anti-PD-1/PD-L1 therapies (36). Results from the CITYSCAPE Phase II trial of anti-TIGIT tiragolumab and atezolizumab in patients with PD-L1+ advanced NSCLC demonstrated grade >=3 TRAE in 15% of patients. ORR was higher in patients receiving tiragolumab and atezolizumab (37.3% (CI 25–49.6) compared to those receiving placebo and atezoliumab (20.6% (CI 10.2–30.9), with an odds ratio of 2.57 (CI 1.07–6.14) (40). Tiragolumab with or without atezolizumab was also tested in patients with advanced solid tumors, in a Phase Ia/Ib dose escalation trial with TRAE of ≥ grade 3 in 4% of patients in each phase (41). There were three responses greater than stable disease in the Phase 1b portion, all of which occurred in PD-L1 positive patients. In an additional NSCLC expansion cohort ORR was 50%. Results are not yet available for additional clinical trials.

**Table 1:** Summary of inhibitory receptors in clinical trials.

Receptor	Binding Partners	Therapies under development	Clinical trials
LAG3	MHC II	IMP321 Relatlimab LAG525 TSR-033 REGN3767 MK-4280	IMP321: Vaccine adjuvant With gemcitabine for pancreatic cancer With paclitaxel in breast cancer With pembrolizumab in HNSCC Relatlimab: With nivolumab in melanoma LAG525: With Spartalizumab in advanced solid tumors
TIM-3	Galectin-9 Ceacam1 HMGB1 Phosphatidylserine	MGB453 TSR-022 Sym023 BGBA425 RO7121661 ICAGN02390 LY3321367 BMS-986258	LY3321367: With and without anti-PD-L1 in advanced solid tumors MBG453: With spartalizumab in NSCLC and melanoma
TIGIT	CD155 CD112	BMS-986207 BGB-A1217 Tiragolumab AB154 ASP8374 MK-7684 COM701 LY3435151	Tiragolumab: With atezolizumab in NSCLC With atezolizumab in advanced solid tumors
VISTA	Unknown	JNJ-61610588 (CI-8993) CA-170 W0180	CA170: In advanced solid tumors and lymphoma In mesothelioma
NRP-1	Class 3 Semaphorins Growth factors (VEGF, TGF, HPF and others)	MNRP1685A ASP1948 CEND1	MNRP1685A: with bevacizumab, with or without paclitaxel in advanced solid tumors CEND1: with gemcitabine and nab-paclitaxel in pancreatic cancer

### Additional Inhibitory Receptors

Additional inhibitory receptors under current clinical investigation include V-domain Ig-containing suppressor of T cell Activation (VISTA) (42). VISTA has homology to CD28 family members including PD-1 (43). Data is primarily available in the preclinical setting, but suggests VISTA blockade may reduce tumor growth in melanoma models, and alter the TME by reducing MDSCs and Tregs (44). The small molecule CA-170 which binds both VISTA and PD-1 has been evaluated in phase I trials with patients with advanced solid tumors, lymphomas and mesotheliomas (45, 46). Phase I trials with anti-VISTA monoclonal antibody is also currently underway.

Neuropilin-1 (NRP1) is another inhibitory receptor under investigation for its clinical potential (47). NRP1 may play a role in T cell dysfunction and is highly expressed on PD1+ intratumoral CD8+ T cells. Murine melanoma models exhibited decreased tumor growth with treatment of combination anti-PD-1 and anti-Nrp-1 (48). Early phase clinical trials with two anti-NRP1 agents were published, but do not appear to have been pursued further, in part due to toxicity (49, 50). An anti-NRP1 monoclonal antibody is currently under clinical development in a Phase 1b trial.

### Co-Stimulatory Receptors

Another strategy has been to target co-stimulatory receptors with monoclonal antibodies that behave as receptor agonists, with or without anti-PD-1 blockade. These receptors include OX40, CD27, 4-1BB, and GITR. Co-stimulatory receptors are present on T cells and counter-act the negative regulation of inhibitory receptors such as PD-1 and CTLA-4 (51). Phase I studies of anti-OX40 agonists have yielded disappointing results, with a single partial response noted in the trial of MEDI0562 in advanced solid tumors, and best response of stable disease with GSK998 (52, 53). When combined with pembrolizumab in a trial enrolling 96 patients, anti-OX40, gave 2 CRs and 7PRs (53). 4-1BB targeted therapy was complicated by hepatic toxicity that was mitigated at lower doses (54). However, efficacy of monotherapy and combination treatment with anti-PD-1 was not particularly impressive (55). Several companies have dropped their pursuit of co-stimulatory monoclonal antibodies, such as OX40 from their pipelines.

## Damage- or Pathogen-Associated Molecular Patterns

Pattern recognition receptors (PRRs) were identified as part of the innate immune system as a first line defense to pathogens. These receptors can recognize pathogen-associated molecular patterns (PAMPs) and include a family of receptors called toll-like receptors (TLRs). Additional receptors have been identified to recognize damage-associated molecular patterns (DAMPs). TLRs are present on both immune and non-immune cell types. Presence of a PAMP/DAMP leads to TLR activation, and downstream activation of transcription factors that result in the production of interferons, and interferon-stimulated responses. In addition to triggering the production of inflammatory cytokines and chemokines, the IFN response is also important for antigen presentation and the priming of an adaptive immune response. Interferon triggers maturation of antigen-presenting cells, leading to presentation of proteins in the context of MHC. This ultimately leads to the induction of specific T and B cell responses. Preclinical work with tumor cell lines and murine models however, have also shown data suggesting TLR stimulation can lead to tumor proliferation (56, 57). Whether the anti-tumor effects of TLR-stimulation can be specifically harnessed is a work in progress.

Although the mechanisms were not known at the time, TLR-agonists were used in early cancer therapy by William Coley (58). In his historic experiments, patients with cancer were injected with lipopolysaccharide (LPS)-containing bacterial concoctions, and tumor regression was occasionally noted. Unsurprisingly, these patients also developed high fevers and other intolerable side effects. LPS was ultimately identified in these bacterial cocktails as the active agent. Since that time many strategies to utilize PRRs in cancer therapy have been exploited as described below and summarized in **Table 2**.

**Table 2:** Toll-like receptor (TLR) agonists under investigation.

Receptor	PAMP	Therapies under development	Clinical trials
TLR3	polyinosinic:polycytidylic acid (poly(I:C))	Rintatolimod ARNAX	Rintatolimod: with IFN for melanoma, colorectal cancer, prostate cancer With pembrolizumab and cisplatin for ovarian cancer With IFN and neoadjuvant chemotherapy for breast cancer
TLR4	Lipopolysaccharide	MPLA GLA-SE GSK1795091	GLA-SE: Vaccine adjuvant GSK1795091: With immunotherapy in advanced solid tumors
TLR7 TLR8	Single-stranded RNA	Imiquimods Resiquimod Motolimod	Imiquimod: With tumor lysate vaccine in grade II gliomas With dendritic cell vaccine in malignant gliomas With dendritic cell and GM-CSF vaccine in ovarian cancer Resiquimod: Vaccine adjuvant Motolimod: With nivolumab in HNSCC With doxorubicin and durvalumab in ovarian cancer
TLR9	Double-stranded DNA	Lefitolimod SD-101 CMP-001 Tilsotolimod	Lefitolimod: With ipilimumab in advanced solid tumors SD-101: With anti-Ox40 for NHL With ibrutinib and radiation for follicular lymphoma With nivolumab and radiation for pancreatic cancer With pembrolizumab in prostate cancer With pembrolizumab in breast cancer CMP-001: With pembrolizumab in HNSCC With Pembrolizumab in melanoma With nivolumab, ipilimumab and radiation in colorectal cancer With immunotherapies in advanced solid tumors

### Toll-Like Receptor Agonists

#### Toll-Like Receptors That Bind Nucleic Acids

TLRs recognizing nucleic acids have been under investigation and promising for some time as therapeutic agents for cancer. These include TLR3, TLR7, TLR8, and TLR9. TLR3 recognizes dsRNA, and a common synthetic nucleic acid used for stimulation is polyinosinic:polycytidylic acid (poly(I:C)). Early studies had issues with toxicity and stability and were discontinued (59). Further, phase I studies with poly(I:C) monotherapy appeared to have little clinical efficacy (60). Since that time, several compounds modified to enhance stability have been produced and are being studied in various malignancies in combination with immunotherapy and as adjuvants in vaccine-based strategies (61). Early phase clinical trials are ongoing with the formulations rintatolimod and ARNAX.

TLR7 and TLR8 recognize single-stranded RNA. Stimulation results in activation of MyD88, and secretion of cytokines, including type I interferons. The synthetic imidazoquinolones have been tested as antiviral treatments, and now derivatives are being explored as cancer therapeutics. Imiquimod is compound used topically to treat several skin conditions, including basal cell carcinomas and is being explored in pre-cancerous lesions, such as cervical intraepithelial neoplasia. Imiquimod has also been used in early phase clinical trials for treatment of cutaneous metastases for breast cancer and melanoma, however has not been pursued further (62, 63). Imiquimods have been explored as an adjuvant cancer vaccines and is being used in a variety of these trials (NCT01678352, NCT00799110, NCT01792505) (64). Resiquimod binds both TLR7 and TLR8 and is similarly being explored as an adjuvant in cancer vaccines (65). Motolimod (VTX-2337) is another imidazoquinolone with preclinical evidence supporting activation of NK cells and priming of CD8+ T cells when given with cetuximab (66, 67). A phase 1b clinical trial of motolimod and cetuximab in patients with head and neck squamous cell carcinoma resulted in demonstration of maximum tolerated dose with 2/13 patients achieving partial responses (68). Studies are ongoing with motolimod in combination with anti-PD1 for patients with head and neck cancer (NCT03906526), as well as with doxorubicin and durvalumab in patients with ovarian cancer (NCT02431559).

TLR9 recognizes double-stranded DNA, typically in the form of unmethylated cytidine phosphate guanosine (CpG) oligonucleotides (ODN) present in pathogens. Several synthetic CpG-ODNs have been investigated for cancer therapy, including in combination with chemotherapy, immunotherapy or as vaccine adjuvants. Lefitolimod (MGN1703) is a DNA molecule tested in a phase I dose escalation with ipilimumab, with planned dose escalation (NCT02668770) (69). SD-101 is another CpG-ODN that has been tested intratumorally in combination with pembrolizumab in a phase 1b clinical trial with an ORR of 15% (70). A number of trials are ongoing evaluating SD-101 in hematologic malignancies, as well as in pancreatic cancer (NCT04050085) and prostate cancer (NCT03007732). CMP-001 is a CpG-A virus-like particle that stimulates TLR9. Intratumoral CMP-001 with pembrolizumab has been shown in a Phase 1b trial demonstrating 24% ORR in melanoma patients who have previously progressed on anti-PD-1 therapy (71). Additional trials are evaluating CMP-001 by subcutaneous administration (NCT03084640), in the neoadjuvant setting (NCT04401995), and in other solid tumors and hematologic malignancies. Tilsotolimod (IMO-2125) is another TLR9 agonist being investigated in combination with nivolumab and ipilimumab for patients with solid tumors (NCT03865082), with preliminary data from a phase 1/2 trial only available by press release thus far.

#### Toll-Like Receptors That Recognize Other Bacterial Products

Additional TLRs target bacterial components. TLR1,2,6, and 4 bind bacterial cell wall products and TLR5 binds flagellin. TLR2 is found on the cell surface and forms a heterodimer with either TLR1 or TLR6 that recognized lipoproteins (72). Although there are several synthetic lipoproteins targeting these receptors being explored in autoimmunity or as adjuvants in therapy of viral infection, none are actively explored in malignancies. BCG is used to treat non-muscle-invasive bladder cancers, and may work in part by inducing inflammation through TLR2 and TLR4 (73). TLR4 agonists have been explored extensively since Coley’s initial observations. An LPS derivative of Salmonella, monophosphoryl lipid A (MPLA) is a TLR4 agonist that is currently used as an adjuvant in the Cervarix HPV vaccine (74). A dose escalation studies of lipid A formulations as monotherapy for were tolerated at lower doses, but did not demonstrate anti-tumor responses as monotherapy (75, 76). TLR4 agonists such as GLA-SE have been used as adjuvants in vaccines directed at malignancies or infectious agents, but are not actively being developed in oncologic trials (77). Currently, GSK1795091 synthetic agonist was tested in healthy volunteers, and a planned trial in combination with immunotherapy in advanced solid tumors is planned (NCT03447314) (78). TLR5 binds flagella, and has been targeted by Mobilan, an adenoviral vector expressing flagellin. Mobilan has been tested in a phase I trial with intratumoral injection for prostate cancer, though has not been pursued further (79).

### Additional Pathogen Recognition Receptors

Additional PRRs have been identified since the initial discovery of TLRs and include RIG-I like receptors (RLRs), NOD-like receptors (NLRs) and C-type lectin receptors (CLRs). The work on these receptors has not been as well-developed as for the TLRs, but are now being explored in cancer immunity. RLRs include two receptors, retinoic acid-inducible gene I protein (RIG-I) and melanoma differentiation associated protein 5 (MDA5) that bind viral RNA molecules. Downstream signaling results in interferon secretion. The RIG-I agonist, MK4621, was tested in a phase I/II trial for advanced solid tumors without dose-limiting toxicities observed (80) and is planned for study in combination with pembrolizumab (NCT03739138). NLRs recognize bacterial products and lead to activation of the inflammasome and IL-1β production. NLR activation may contribute to carcinogenesis, and is being explored for potential therapeutic targets (81, 82). CLRs are a large group of PRRs that bind a wide variety of ligands and can result in pro-inflammatory and anti-inflammatory responses, and are trying to be understood (83). STING is an additional nucleic acid PRR located in the endoplasmic reticulum (84). Many early phase clinical trials are underway with STING agonists as monotherapy, in combination with immunotherapy, chemotherapy, or radiation (85).

## Immune Signaling Mediators

### Cytokines

Cytokines in the TME are produced by infiltrating tumor and stromal cells, and can contribute to either a pro-inflammatory or anti-inflammatory milieu (86). Cytokine therapy has FDA approved indications with IL-2 for renal cell carcinoma and melanoma, and type I interferon for adjuvant therapy of melanoma, and in CML and MPNs. While these therapies are used infrequently in melanoma in favor of current ICI immunotherapy, a small number of patients do well, and may have been cured with IL-2 in metastatic RCC (87). GM-CSF has also been investigated for its potential benefits in cancer therapy. The GM-CSF modality is currently an underlying basis of the benefits of talimogene laherparepvec, an FDA-approved oncolytic immunotherapy for melanoma (88). The body of literature discussing the many functions of cytokines in cancer therapy is vast, and this review will focus on those therapies currently under development.

Toxicity has been a significant issue with cytokine administration in clinical trials and standard therapies, as evidenced by the experience with IL-12. Preclinical data demonstrated the anti-tumor effects of IL-12 by multiple mechanisms and in several murine tumor models (89). However, although a dose was selected in phase I trials to minimize toxicity, the phase II study was halted after significant toxicity and 2 patient deaths (90, 91). The additional toxicities seen in the phase II trial were thought to be a result of a change in the dosing schedule, when a priming initial dose was no longer given. Interest in IL-12 persists, with formulations such as NHS-IL12, an IL12 heterodimer fused to an antibody, tested in a phase I trial (92). A number of ongoing trials with this compound are ongoing, as monotherapy and in combination with immunotherapy. Additional studies use IL-12 expressing viral or CART constructs.

IL-15 shares part of the IL-2 receptor and signaling pathway, and similarly results in NK and T cell proliferation (93). Unlike IL-2, however, IL-15 is not thought to stimulate T regulatory cells, making it an attractive target. Recombinant IL-15 (rhIL15) has been studied in patients with advanced solid tumors in a phase I study, notable for increased NK and CD8+ T cell proliferation seen in the peripheral blood (94). Subcutaneous rhIL15 along with haploidentical NK cell infusion was used to treat patients with acute myeloid leukemia, demonstrating NK cell proliferation and 40% remission rates, though cytokine release syndrome was noted in 56% of these patients (95). ALT-803 is a IL-15/IL-15Ra Fc fusion complex, referred to a superagonist, that has been tested in PD-1 refractory NSCLC patients evaluated in phase Ib study, with additional studies ongoing (96). Other formulations of IL-15 have been developed and are undergoing clinical trials, including recombinant proteins BJ-001, PF-07209960, NIZ985, and N-803. Additional combinations of IL-15 products are being conducted with immunotherapy, and as part of adoptive cell therapy products.

Interferon-γ is a type III interferon potently induced by IL-12, whose expression is associated with immunotherapy response in melanoma (8). However, IFNγ has been associated with both anti-tumor and pro-tumor effects (97). Clinical trials with IFNγ as monotherapy have not been fruitful, perhaps due to its seemingly contradictory role in the TME. Other cytokines initially pursued and since abandoned for toxicity and lack of efficacy include IL-21 and IL-7 (98).

### Small Molecule Inhibitors

In addition to traditional cytokines and chemokines, the TME also contains a number of small molecules that effect the inflammatory state of the tumor. Indoleamine 2,3-dioxygenase 1 (IDO1) helps convert tryptophan to kynurenine, which has an immunosuppressive effect on the TME. Kynurenine promotes development of Tregs and MDSCs (99). Epacadostat is an IDO1 inhibitor that has been studied in combination with pembrolizumab. Although the initial phase I/II trial in advanced solid tumors showed promise, the phase III trial in melanoma did not show a difference in progression-free survival or overall survival versus placebo with pembrolizumab (100, 101). Many of the ongoing trials of IDO1 inhibitors have since been terminated, though a few trials with IDO1 inhibitor BMS-986205 are still recruiting.

The phosphoinositide 3-kinase (PI3K) signaling pathway functions at many stages of cancer biology including cell division, differentiation, motility and metabolism (102). Inhibitors downstream of the PI3K pathway are active in some solid tumors, including everolimus in neuroendocrine tumors (103) and everolimus with exemestane in breast cancer (104). Targeting the PI3K isoforms γ and δ, that are specifically expressed in hematopoietic cells, is an area of investigation supported by preclinical work showing alterations in the TME to a pro-inflammatory phenotype (105). PI3Kγ and γ/δ inhibition are being studied in clinical trials.

## Concluding Remarks

The strategies discussed in this review highlight a number of promising approaches for overcoming immunotherapy resistance, a significant treatment dilemma for patients with advanced melanoma. These therapies all aim to increase local inflammation in the TME but by drastically different mechanisms, with varying routes of administration and toxicities. Of these, use of DAMPs/PAMPs are of particular promise, and early phase trials have shown intratumoral and administration with anti-PD-1 to be tolerable with early signs of efficacy. Development of intravenous formulations or formulations compounded with anti-PD-1 monoclonal antibodies could be an interesting avenue to explore. We look forward to more data in this field and with other tumor types.

One major challenge in the development and testing of novel immunotherapeutics is the heterogeneity of mechanisms of resistance. Patients have varying expression of inhibitory receptors after immunotherapy, differing levels and types of immune infiltrates, and differences in mutational profiles and epigenetic changes that can all alter response to immunotherapy (106). This heterogeneity may also contribute to the limited efficacy observed in some trials. However, without definitive biomarkers to reliably sort patients by mechanism, this truly personalized approach remains currently out of reach. While some biomarkers are certainly helpful, such as TMB and PD-L1 expression, even these do not always correlate with response (107).

Ongoing efforts to identify biomarkers are underway, including with gene-expression profiling, such as those signatures associated with IFNγ (8, 108). Until robust biomarkers are identified and then correlated with response to specific therapies, an all-comers approach must be utilized. After validation of a biomarker-based treatment approach, these therapies could also be explored in the front-line setting, perhaps identifying those at risk for primary resistance to immunotherapy, and ultimately leading to greater portion of those with durable responses. Cost-benefit analysis would also be an important factor in the design of a biomarker-driven, personalized medicine approach to avoid contributing to the already egregious cost of oncologic care that may benefit a small portion of patients.

The strategies discussed in this review are only a part of the approach being considered for overcoming immunotherapy resistance, and a number of other promising strategies that are under development. These include vaccine development with tumor-associated antigens, in part with the adjuvants mentioned here. Other strategies that may in the near future gain regulatory approval include adoptive cell transfer, both with the use of TILs and perhaps with CAR-T therapies. Finally, targeting other elements of the TME that are a more fundamental basis of immunotherapy resistance, such as myeloid derived suppressor cells and T regulatory cells are also under development. There is much reason for excitement given the breadth and pace of development of immunonotherapeutics and forthcoming results in the next several years will dictate the future of the field.

## Author Contributions

AR contributed to the design, concept, research, writing, and critical review of this review. JK contributed to the design, concept, critical review, and supervision of this review. All authors contributed to the article and approved the submitted version.

## Funding

The work of the authors is supported by the NIH National Cancer Institute SPORE in Skin CAncer grant P50 CA-121973.

## Conflict of Interest

JK has received grants and personal fees for consultancy from Amgen, Bristol-Myers Squibb, Checkmate and Novartis, and grants from Castle Biosciences, Immunocore, and Iovance.

The remaining author declares that the research was conducted in the absence of any commercial or financial relationships that could be construed as a potential conflict of interest.
